# Identification and association mapping of sources of stem rust resistance in the wild barley diversity panel effective against virulent isolates from the Pacific Northwest

**DOI:** 10.1093/g3journal/jkaf300

**Published:** 2025-12-20

**Authors:** Matthew Brooke, Arjun Upadhaya, Shaun J Clare, Karl Effertz, Robert Brueggeman

**Affiliations:** Department of Crop and Soil Sciences, Washington State University, Pullman, WA 99161 United States; Department of Crop and Soil Sciences, Oregon State University, Corvallis, OR 97330 United States; Department of Crop and Soil Sciences, Washington State University, Pullman, WA 99161 United States; Department of Plant Pathology, Washington State University, Pullman, WA 99161 United States; Department of Crop and Soil Sciences, Washington State University, Pullman, WA 99161 United States; Department of Crop and Soil Sciences, Washington State University, Pullman, WA 99161 United States; Department of Crop and Soil Sciences, Washington State University, Pullman, WA 99161 United States

**Keywords:** stem rust, barley, genome-wide association study

## Abstract

Identifying and genetically characterizing new sources of resistance in barley (*Hordeum vulgare*) effective against the virulent wheat stem rust (*Puccinia graminis* f. sp. *tritici* [*Pgt*]) population in the Pacific Northwest (PNW) is critical. Isolates from this population, including *Pgt* isolate Lsp21, were virulent on barley stem rust resistance (R) genes *Rpg1*, *Rpg2*, *Rpg3*, *rpg4*, *Rpg5*, and *rpg8*. Notably, 10% of the *Pgt* isolates from the population were virulent on barley line Q21861, which contains *Rpg1* and *rpg4/5* stacked together. Virulence on these 2 broad and effective stem rust *R*-genes/loci, when combined, is unprecedented *Pgt* virulence on barley. To discover novel resistance, 277 wild barley (*H. vulgare* subsp. *spontaneum*) accessions from the Wild Barley Diversity Collection (WBDC) were screened with *Pgt* isolate Lsp21. Twelve percent showed moderate resistance, with WBDC-94 and WBDC-238 from Jordan exhibiting exceptional resistance, likely conferred by *Rpg7*, previously reported in both. To genetically characterize resistance in the WBDC, a genome-wide association study was conducted using disease reactions to Lsp21 and 37,338 genotyping-by-sequencing SNPs. Twelve resistance-associated loci were identified on chromosomes 1H, 2H, 3H, 5H, 6H, and 7H. *Rpg7* was not detected due to its low allele frequency in the panel. Importantly, 7 novel resistance loci, WQ*Rpg*-2H01, WQ*Rpg*-2H02, WQ*Rpg*-3H01, WQ*Rpg*-5H01, WQ*Rpg*-5H03, WQ*Rpg*-7H02, and WQ*Rpg*-7H03, were discovered. These new sources of resistance can be integrated into cultivated barley, and the associated SNPs will aid in tracking resistance loci in prebreeding lines, enhancing breeding efforts against the virulent PNW *Pgt* population.

## Introduction

Wheat stem rust (*Puccinia graminis* f. sp*. tritici* [*Pgt*]) is a significant foliar disease of barley (*Hordeum vulgare* L.) and wheat (*Triticum aestivum* L.). In 2019, barley stems with susceptible stem rust pustules containing urediniospores were collected from production fields and experimental plots in eastern Washington. Single spore isolates (*n* = 100 isolates) were generated, providing a representation of the isolates present in this wheat stem rust population (Upadhaya et al. 2022). Phenotyping assays using a barley stem rust differential set determined that 99% of the 100 isolates were virulent on the cultivar “Morex” (CIho 15773), which contains the *Resistance to Puccinia graminis 1* (*Rpg1*) gene, 16% of the isolates were virulent on the line HQ1 containing the *rpg4*/*Rpg5*-mediated resistance locus (RMRL), and 10% of the isolates were virulent on barley line Q21861 (PI 584766), which contains both *Rpg1* and *RMRL.* When *Rpg1* and RMRL are stacked together, they provided exceptionally broad resistance that was effective against all known races and isolates of *Pgt* collected from around the globe. Thus, this remarkable virulence is the first documentation of *Pgt* virulence on the *Rpg1* and RMRL gene combination, and this Pacific Northwest (PNW) stem rust population represents the most virulent population of stem rust reported on barley (Upadhaya et al. 2022, 2024).

*Pgt* is a heteroecious biotrophic fungal pathogen with a complex life cycle producing 5 distinct spore stages on 2 unrelated hosts. The primary monocot hosts are wheat, barley, and wild grasses, and the secondary hosts are common barberry (*Berberis vulgaris*) and mahonia (*Mahonia* spp.) (Roelfs 1985). As the primary cereal and grass hosts mature in late summer and senesce, the fungal pathogen transitions from producing urediniospores to teliospores. These teliospores can overwinter in moderate climates to specifically infect their dicot secondary hosts, common barberry or mahonia, in the spring. During the completion of its sexual cycle on secondary dicot hosts, aeciospores containing recombinant gametes form and disseminate, infecting primary grass hosts. This can result in the evolution of new virulence gene combinations and serve as early-season inoculum on cereal hosts. This early-season inoculum can initiate the asexual polycyclic disease, where cereal crops are continually infected with urediniospores throughout the summer.

In the early 1900s, the Midwestern United States (US) and the Prairie Provinces of Canada experienced major stem rust epidemics (Dyck and Kerber 1985). During that time, exceptionally bad disease years resulted in nearly 100% crop loss for growers. These severe epidemics occurred when environmental conditions were conducive to disease formation and susceptible varieties were grown across vast acreage. Early-season infection initiated by inoculum originating from the secondary host common barberry growing near wheat and barley fields was especially problematic as earlier infections can result in more severe epidemics. Thus, it was observed that the removal of this source of inoculum was an effective disease management strategy. The earliest record of barberry eradication dates back to a law in Rouen, France, in 1660, which advocated for the destruction of barberry plants to protect cereal crops (McKay 1957). Common barberry was introduced to North America by European settlers in the 1800s and became a widespread invasive species (Stakman and Fletcher 1930; Hill 2003). Years later, in 1918, the US Federal Government implemented the barberry eradication program (Roelfs 1982) to eliminate the threat posed to wheat and barley production in the US. Over the next half-century, the barberry eradication program effectively eliminated susceptible barberry bushes, thereby stopping the *Pgt* sexual cycle and the contribution of early-season inoculum. With the stabilization of the stem rust population in the Midwestern US and deployment of effective resistance genes in wheat and barley, major stem rust epidemics became a thing of the past (Roelfs 1978; Maloy 1993), and the federal barberry eradication program was discontinued in 1977.

It was recently discovered that the PNW region of North America has become the center of stem rust diversity on the continent due to the endemic presence of mahonia, another secondary sexual host of *Pgt* (Jin et al. 2014; Upadhaya et al. 2024). Mahonia is native to the PNW and found widespread throughout woodland areas as part of the natural ecosystems; thus, eradication is not possible. Climate change also contributes to the completion of the disease cycle as warmer winters and increased winter and spring precipitation in the PNW contribute to an environment more conducive for infection of both the primary and secondary hosts. The completion of the sexual cycle gives rise to greater diversity and contributes to the evolution of new virulent gene combinations and races. This poses a threat to North American cereal production as these races disseminate to other regions of the continent. This occurred with the *Pgt* race QCCJB, which emerged in the upper Great Plains of North America during the 1990s, exhibiting unprecedented virulence against the barley stem rust resistance gene *Rpg1*. A recent study indicates that QCCJB originated from the PNW sexual *Pgt* population (Upadhaya 2023).

Novel sources of stem rust resistance effective against this virulent PNW population have been identified using 440 diverse accessions from the World Barley Core Collection (WBCC) (Upadhaya 2023; Brooke et al. 2025). The WBCC is an extensive collection of over 18,000 diverse domesticated barley (*H. vulgare* subsp. *vulgare*) lines collected from around the world, including landraces and cultivated food, feed, and malting barley accessions. Upadhaya (2023) utilized SNP data generated for ∼1,500 WBCC lines to organize a “mini” core collection that represented the diversity present in the collection. Based on the mini core collection, a genome-wide association study (GWAS) was conducted using 440 diverse lines from the WBCC to screen for seedling resistance against *Pgt* isolate Lsp21, the most virulent PNW isolate on barley. Of the 440 lines screened, only 6% were resistant, and 3 marker–trait associations (MTAs) were detected on chromosomes 3H, 5H, and 6H.

To identify additional resistance genes/loci effective against the PNW stem rust population, the Wild Barley Diversity Collection (WBDC) was screened and genetically characterized using GWAS. The WBDC is a collection of wild barley (*H. vulgare* subsp. *spontaneum*) comprising 318 accessions collected from the Fertile Crescent, Asia, North Africa, and the Caucasus region (Steffenson et al. 2007). Cultivated barley was domesticated over 10,000 years ago from the wild barley progenitor *H. vulgare* subsp. *spontaneum* in the Fertile Crescent region (von Bothmer et al. 2003). However, much of the diversity in wild barley was lost during domestication and selection of *H. vulgare* (Wambugu et al. 2018). *H. vulgare* subsp. *spontaneum* is a rich source of genetic diversity for numerous traits, including abiotic and biotic stress resistances (Ellis et al. 2000; Fetch et al. 2003; Steffenson et al. 2007; Sallam et al. 2017; Liu et al. 2020; Henningsen et al. 2021; Clare et al. 2023). Therefore, *H. vulgare* subsp. *spontaneum* serves as a reservoir of stem rust resistance sources because both the host and the pathogen have been coevolving in a host–pathogen molecular arms race in the Fertile Crescent for thousands of years (Fetch et al. 2003).

The goal of this research was to identify novel sources of seedling resistance against the virulent PNW population of *Pgt*. Only 8 stem rust resistance loci have been formally designated in barley: *Rpg1*, *Rpg2*, *Rpg3*, *rpg4*, *Rpg5*, *rpg6*, *Rpg7*, and *rpg8* (Steffenson et al. 2017; Henningsen et al. 2021; Matny et al. 2024). The *rpg8* gene was formerly known as *rpgBH* but was recently genetically characterized and given the *rpg8* gene nomenclature (Matny et al. 2024). The virulent *Pgt* isolates from the PNW population have been shown to be virulent on *Rpg1*, *Rpg2*, *Rpg3*, *rpg4*, *Rpg5*, and *rpg8* (Upadhaya et al. 2022). Because of the threat posed by the virulent population of stem rust in the PNW, new barley resistance sources and genes must be identified and characterized. We turned to wild barley because it has coevolved with stem rust in the Fertile Crescent and could contain novel sources of resistance effective against the PNW *Pgt* population.

## Materials and methods

### Plant material and pathogen

This GWAS study utilized the WBDC, comprising 318 *H. vulgare* subsp. *spontaneum* accessions obtained from the United States Department of Agriculture (USDA) National Small Grains Collection, Aberdeen, Idaho (Supplementary Table 1). These accessions were collected from the Fertile Crescent, Central Asia, North Africa, and the Caucasus region, where *H. vulgare* subsp. *spontaneum* is native (Steffenson et al. 2007). The domesticated barley (*H. vulgare* L) varieties Morex (CIho 7124), Steptoe (CIho 15229), and Q21861 (PI 584766) were used as susceptible checks, while Elliot (PI 592261) and DH-160748 were used as resistant checks. Morex is a 6-row malting line that contains *Rpg1.* Q21861 is a 2-row barley line that contains *Rpg1* and the RMRL. Steptoe is a 6-row feed line developed by the Washington State University breeding program that has no known wheat stem rust *R*-genes. Elliot is a 2-row malting variety developed in the United Kingdom, derived from a “Trumpf” × “Hassan” cross in 1993, which contains moderate resistance effective against PNW isolate Lsp21 (Brooke et al. 2025). DH-160748 is a double haploid experimental malt line from the Oregon State University (OSU) barley breeding program that contains uncharacterized resistance effective against *Pgt i*solate Lsp21.

The PNW *Pgt* isolate Lsp21 was selected for phenotyping because it represents the most aggressive and virulent isolate on barley from the recently characterized PNW population. This was determined by infection type (IT) assays on a barley stem rust *R*-gene differential set (Upadhaya et al. 2022). *Pgt* isolate Lsp21 was virulent on barley lines Morex (*Rpg1+*), CIho 7124 (*Rpg2+*), PI282313 (*Rpg3+*), HQ1 (*rpg4*/*Rpg5+*), Q21861 (*Rpg1+* and *rpg4*/*Rpg5+*), and “Black Hulless” (*rpg8+*) (Supplementary Table 2). This virulence profile on barley *R*-genes represents the most virulent isolates of *Pgt* reported on barley.

### Plant growth conditions

For the seedling disease reaction analysis, a single seed from each WBDC accession, as well as the susceptible and resistant checks, was planted in 3 individual 98-well cone containers (6.5 cm diameter by 26.5 cm height). Each cone was filled with standard potting mix soil (Sun Gro Horticulture, Agawam, Massachusetts, United States) supplemented with 2 gm per cone of slow-release Osmocote 14-14-14 fertilizer. Plants were placed in a growth chamber set to 18 °C with a 16 h (400 μm/m^2^) light and 8 h dark cycle as described by Upadhaya et al. (2022). Furthermore, the experiment was replicated 2 times in a complete randomized design. Accessions with variable reactions across replicates were phenotyped for an additional replication.

### Stem rust inoculations and incubations

Approximately 9 d after planting, when the primary leaves were fully expanded, stem rust inoculations were conducted using an atomizer pressured by an air pump set at 30 kPa (Steffenson et al. 2017; Upadhaya et al. 2022). Seedlings were inoculated with fresh urediniospores (collected from Steptoe seedlings inoculated with *Pgt* isolate Lsp21) and mineral oil at 8 mg of urediniospores per 1 ml of mineral oil. After inoculation, plant leaves were allowed to dry for 1 h and then placed in a mist chamber for 18 h in complete darkness at 18 °C and 100% relative humidity. After 18 h, plants were placed back in the growth chamber at the conditions previously described.

### Stem rust scoring

At 14 d after inoculation, ITs were assessed on primary leaves. Infections were rated on a modified “0 to 4” scale. This scale was initially developed by Stakman et al. (1962) for wheat, later modified for barley by Miller and Lambert (1955), and further modified by Steffenson et al. (2017). When conducting stem rust analysis on barley, mesothetic reactions of different ITs on the same primary leaf can be observed. These IT scores were categorized as 0; = hypersensitive reaction (HR), 1 = resistance (R), 2 = moderately resistant, 3- = moderately susceptible, and above a 3 was considered susceptible (Steffenson et al. 2017; Hernandez et al. 2019). For more accurate estimation of pustule size, + and − symbols were used after the corresponding IT. The categorical IT scores of “0 to 4” were converted into numeric coefficient of infection (CI) values (Zhou et al. 2014) providing quantitative scores of 0 to 5 that could be utilized in the association mapping analyses. When multiple IT scores were observed on a single leaf, the CI was calculated by order of frequency using a weighted average (Zhou et al. 2014).

### Genotype data

The WBDC had been previously genotyped using a restriction site-associated DNA-genotyping-by-sequencing (RAD-GBS) approach (Sallam et al. 2017). These genotyping data were submitted to the T3 barley database under the project name 2016 GBS_WBDC (https://triticeaetoolbox.org/). The genotyping data were downloaded from the T3 barley database in the vcf file format containing 50,842 SNP markers with approximately 8.6% of missing genotypic data. Imputation of missing SNP marker data was completed using the Beagle 5.4 software (Pook et al. 2020). Due to the nature of wild barley, the heterozygous SNP calls were included in the final analysis. SNPs with minor allele frequency (MAF) < 0.05% were removed from the data to address the possibility of detecting false-positive MTAs due to MAF. Linkage disequilibrium (LD) decay was calculated in TASSEL, resulting in 37,338 total SNP markers. The final SNP marker density plot and QQ plots are shown in Supplementary Figs. 1 and 2.

### GWAS analysis

The association mapping analysis was performed utilizing GAPIT v3 in R, with the Bayesian-information and Linkage-disequilibrium Iteratively Nested Keyway (BLINK) model (Huang et al. 2019; Wang and Zhang 2021). Seven principal components (PCs) were chosen in GAPIT and incorporated into the GWAS analysis to account for population structure (Lipka et al. 2012). A Bonferroni adjustment was applied at an *α*-level of 0.05 to avoid type I errors. MTAs from the GWAS were considered significant at a *P*-value < 0.00000134, corresponding to a logarithm of odds (LOD) (−log_10_[*P*-value]) score > 5.87. After using the BLINK model, a Manhattan plot was generated using the “CMplot” package in R to visualize significant MTAs (Yin et al. 2021).

### Candidate gene identification

The physical positions of the GBS markers were initially aligned to the Morex v1 genome assembly (Beier et al. 2017; Sallam et al. 2017) using the GrainGenes browser (https://wheat.pw.usda.gov/GG3/). The flanking sequence (∼200 bp) from the Morex v1 assembly of each SNP that was identified as a significant MTA was aligned to the Morex v3 genome assembly using the BLAST function to identify physical positions on the current barley genome assembly (Mascher et al. 2021). The genotype data in hapmap format were converted to plink format files using the TASSEL v5.2.93 software (Bradbury et al. 2007). The LD blocks were estimated in Haploview software using the solid spine of LD (Barrett et al. 2005).

Candidate gene models annotated in the Morex v3 genome assembly were used in BLASTp searches on NCBI (https://blast.ncbi.nlm.nih.gov/Blast.cgi) to validate predicted gene function given in the Morex v3 assembly as some predicted gene functions or known domain identities have been misannotated on the Morex v3 genome annotations (Blum et al. 2021). Nonsignificant flanking markers outside the calculated LD blocks were used to delimit the region of the MTA. Nomenclature for each MTA is as follows: *WQRpg1H*-1, where W = wild barley, Q = quantitative trait loci (QTL), and *Rpg = Resistance to Puccinia graminis*, followed by chromosome designation and number of MTAs. Loci were considered novel if they did not overlap with other previously reported QTL. If no genes were identified within the QTL using the Morex v3 assembly, the nonsignificant flanking markers were used to determine the region. Next, the sequences from nonsignificant flanking markers were located in the 9 WBDC accessions within the Pangenome v2 using GrainGenes BLAST service (https://graingenes.org/blast/) to determine the differences in gene content between Morex and WBDC accessions in the barley pangenome (Yao et al. 2022; Jayakodi et al. 2024).

## Results

### Phenotypic observations

**Fig. 1.**
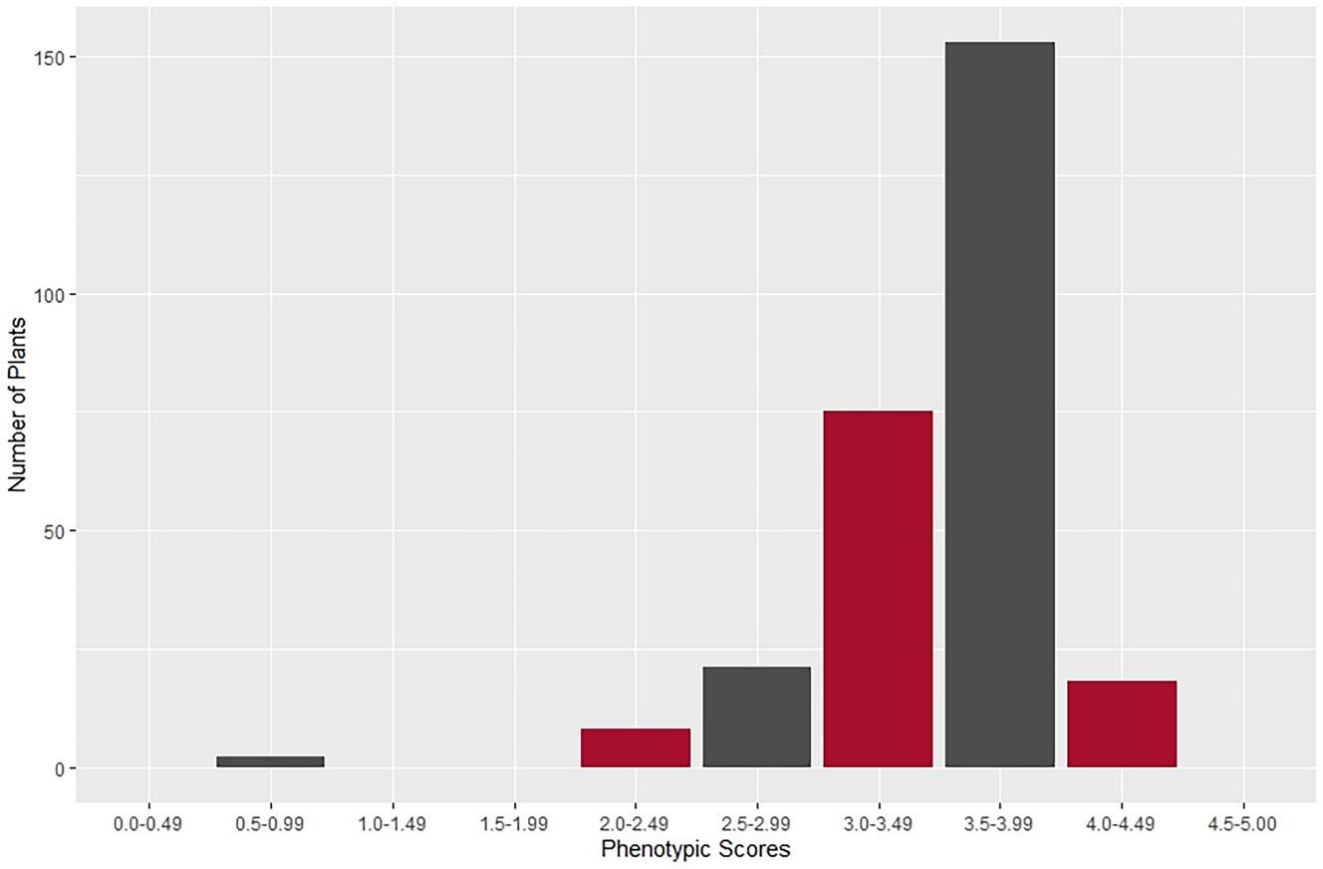
Phenotypic distribution of CI scores of 277 WBDC accessions using *Pgt* isolate Lsp21 from the PNW.

**Fig. 2.**
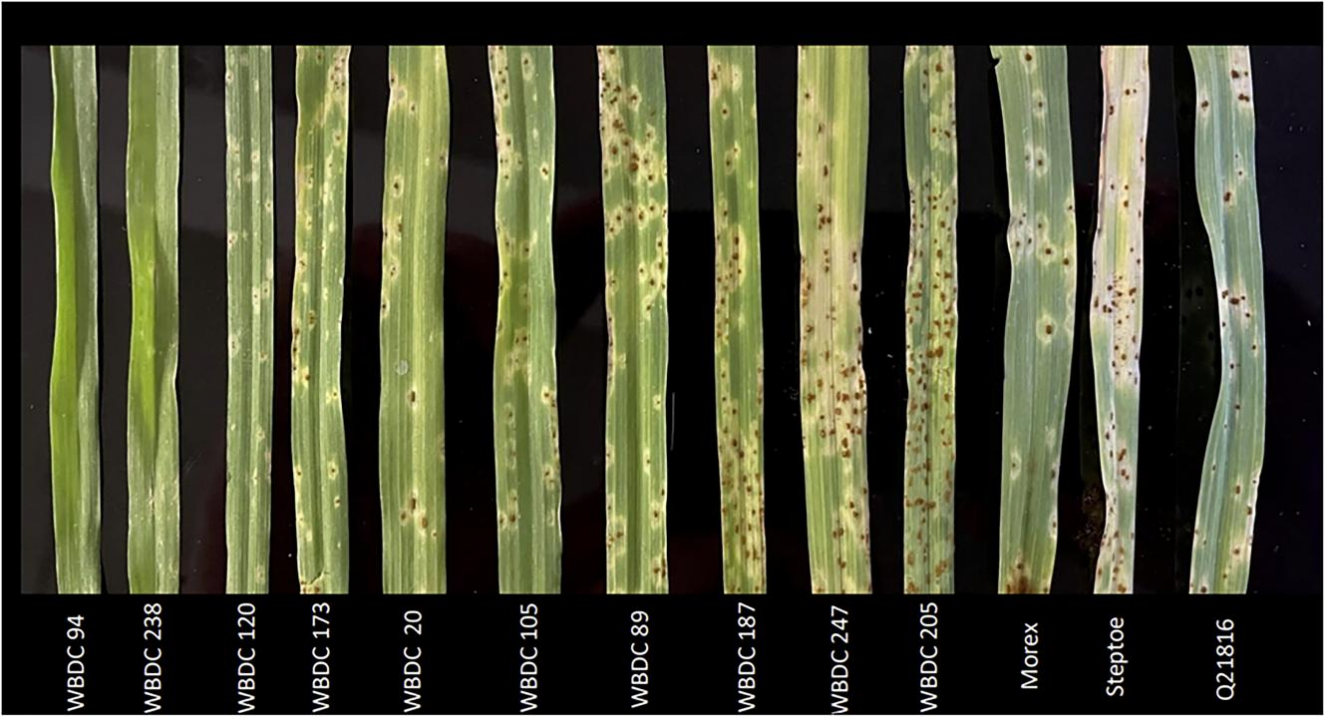
Seedling stage stem rust assay on barley from the WBDC against *Pgt* isolate Lsp21 at 14 d after inoculation. The panel shows a typical disease assay with the virulent *Pgt* isolate Lsp21. This isolate is virulent on the important resistance genes *Rpg1* and RMRL when they are stacked together, Morex (*Rpg1+*) and Q21861 (*Rpg1+* and *RMRL+*).

Of the original 318 accessions within the WBDC (Steffenson et al. 2007), 277 were phenotyped (Supplementary Table 3). The accessions that were not phenotyped (*n* = 41) were excluded due to poor, inconsistent, or delayed germination. Of the 277 accessions phenotyped, only 22 were resistant or moderately resistant, with CI scores of <2.75. The susceptible checks, Morex, Steptoe, and Q21861, were all susceptible, with CI scores averaging 3.33, 3.19, and 2.92, respectively (Table 1). The resistant checks Elliot and DH-160748 were moderately resistant, with CI scores averaging 2.86 and 2.75. The average CI score for all 277 WBDC accessions was 3.51, which was skewed toward susceptibility, with a maximum CI score of 4.41 and a minimum of 0.56 (Fig. 1). The 2 lines WBDC-94 (PI 681809) and WBDC-238 (PI 681943) displayed a strong resistant response to the virulent *Pgt* isolate Lsp21 and were the 2 lines with CI scores of 0.56 (Fig. 2). Both of these accessions were collected near Mādabā, Jordan, and were previously reported as having an HR response to *Pgt* races QCCJB, MCCFC, HKHJC, and rye stem rust (*P. graminis* f. sp. *secalis* [*Pgs*]) isolate 92-MN-90 at the seedling stage (Table 1) (Sallam et al. 2017). Furthermore, the 12 other accessions that displayed moderate resistance to *Pgt* isolate Lsp21 were also resistant to other races of *Pgt* and *Pgs* (Table 1). However, excluding WBDC-238 and WBDC-94, none of the other top resistant accessions to Lsp21 were resistant to *Pgt* race HKHJC.

**Table 1.** Summary of the median infection type (IT-M), range (IT-R), and average CI on the resistant–moderately resistant barley accessions from the WBDC against the PNW stem rust isolate Lsp21.

Name^a^	Accession	IT-M^b^	IT-R^c^	CI^d^	Accessions resistant to *Pgt* and *Pgs* races (Sallam et al. 2017)
WBDC-094	PI 681809	0;	0; to ;1	0.56	QCCJB, HKHJC, and 92-MN-90
WBDC-238	PI 681943	0;	0; to 0;1	0.56	QCCJB, MCCFC, HKHJC, and 92-MN-90
WBDC-123	PI 681833	12;	1; to 12;	2.09	
WBDC-157	PI 681866	21;	;12 to 21;	2.19	QCCJB
WBDC-120	PI 681830	21;	0;1 to 21;	2.23	QCCJB and 92-MN-90
WBDC-173	PI 681880	21;	1;2 to 2	2.25	
WBDC-330	PI 682022	21;	1;2 to 23-1	2.40	
WBDC-213	PI 681919	21	1;2 to 2	2.46	TTKSK, QCCJB, and MCCFC, 92-MN-90
WBDC-214	PI 681920	21	1;2 to 2	2.47	TTKSK, QCCJB, and MCCFC, 92-MN-90
WBDC-020	PI 681744	21	21; to 2	2.60	
WBDC-243	PI 681947	21	21; to 213-	2.63	QCCJB
WBDC-119	PI 681829	12;	12; to 213-	2.64	TTKSK, QCCJB, and MCCFC, 92-MN-90
WBDC-305	PI 682003	21	21; to 3-2	2.65	
WBDC-013	PI 681737	21;	21; to 23-	2.69	QCCJB and 92-MN-90
WBDC-002	PI 681727	21	21 to 2	2.75	
WBDC-017	PI 681741	21	21 to 213-	2.75	QCCJB
WBDC-246	PI 681950	21;	21; to 23-	2.80	QCCJB
WBDC-105	PI 681817	2	21 to 2	2.83	
WBDC-260	PI 681962	21	21 to 2	2.85	QCCJ and 92-MN-90
WBDC-170	PI 681877	21	21 to 2	2.88	QCCJB and 92-MN-90
WBDC-209	PI 681915	213-	213- to 3-	2.88	TTKSK, QCCJB, and MCCFC, 92-MN-90
WBDC-137	PI 681847	3	21; to 3	2.89	
Morex	CIho 15773	3-2	23- to 3-	3.33	
Steptoe	CIho 15229	3-2	21 to 3-2	3.19	
Q21861	PI 584766	23-	21- 23-	2.93	TTKSK, QCCJB, HKHJC, and MCCFC
Elliot	PI 592261	21	21; to 23-	2.86	
DH-160748	-	21	21 to 21	2.75	

Resistant (Elliot and DH160748) and susceptible (Morex, Steptoe, and Q21861) checks are also included.

^a^Top resistance accessions from the WBDC against PNW isolate Lsp21.

^b^IT-M represents the most common or frequent IT observed in multiple experiments (2 to 3). ITs were based on the modified 0 to 4 rating scale initially developed for wheat by Stakman et al. (1962) and modified based on uredinia sizes on barley as described by Miller and Lambert (1955).

^c^IT-R represents the range of ITs.

^d^The CI represents a numeric, quantitative disease score of 0 to 5, calculated from a categorical phenotype score of 0 to 4, as described by Zhou et al. 2014. CI values are means across experiments.

### MTAs and candidate genes

**Fig. 3.**
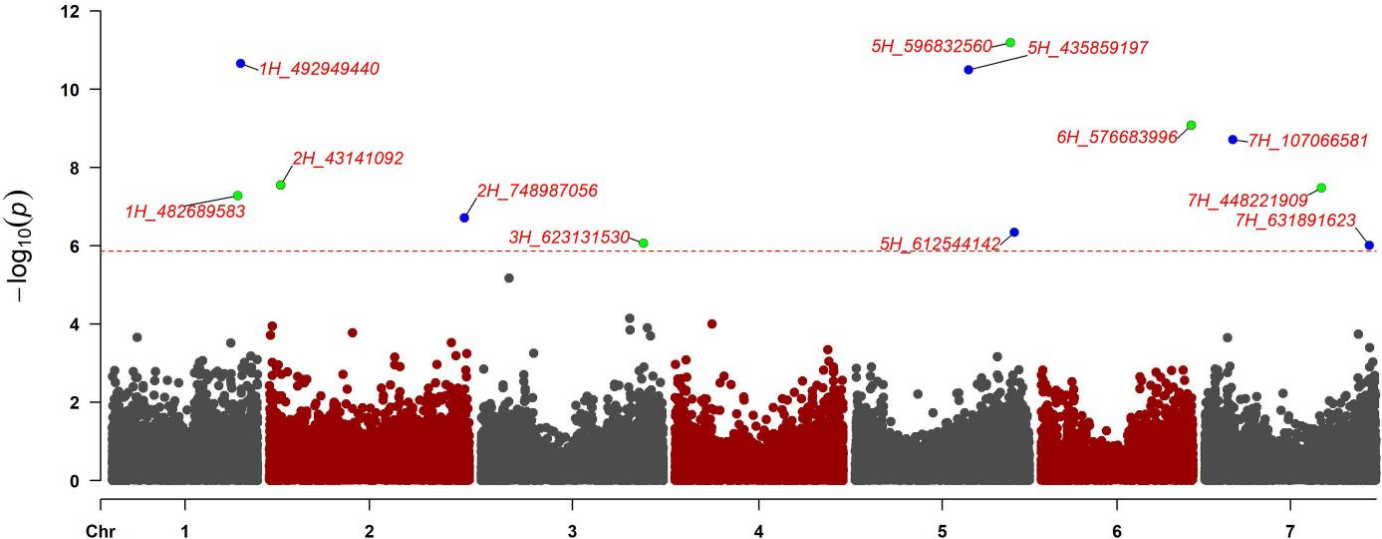
Manhattan plot displaying the 7 chromosomes of barley labeled below. SNPs are shown distributed across the chromosome based on positions on the cv Morex v1 genome assembly. The 12 MTAs associated with resistance to *Pgt* isolate Lsp21 are above the significance threshold (horizontal dashed line) and labeled according to their genome positions on each chromosome based on the cv Morex v1 genome assembly.

The BLINK model identified 12 significant MTAs for resistance to *Pgt* isolate Lsp21 spread across the genome on barley chromosomes 1H, 2H, 3H, 5H, 6H, and 7H (Fig. 3 and Supplementary Table 4). The 12 significant markers had a phenotypic variation explained (PVE) by individual markers ranging from 0.55% for 2H_478987056 to 36.5% for 5H_596832560 (Table 2 and Supplementary Table 4). Nonsignificant flanking markers outside the calculated LD blocks were used to delimit the regions of each MTA that may contain candidate resistance genes underlying each locus (Supplementary Table 5).

**Table 2.** Markers significantly associated with seedling resistance in the WBDC to the PNW *Pgt* isolate Lsp21 using the BLINK model in GAPIT.

Loci	Marker	Allele^a^	Chr^b^	Position^c^	LOD^d^	MAF^e^	PVE^f^	LD decay (kbp)
WQ*Rpg-*1H01	1H_482689583	C/**G**	1H	449,965,275	7.28	0.20	0.96	221
WQ*Rpg-*1H02	1H_492949440	C/**T**	1H	459,175,825	10.66	0.05	6.61	592
WQ*Rpg-*2H01	2H_43141092	G/**A**	2H	37,171,396	7.55	0.08	1.63	1,041
WQ*Rpg-*2H02	2H_748987056	**T**/C	2H	649,620,603	6.72	0.47	0.55	116
WQ*Rpg-*3H01	3H_623131530	C/**G**	3H	555,008,110	6.07	0.05	2.00	20
WQ*Rpg-*5H01	5H_435859197	G/**C**	5H	388,581,717	10.49	0.23	2.31	2,097
WQ*Rpg-*5H02	5H_596832560	G**/A**	5H	526,215,093	11.19	0.06	36.50	133
WQ*Rpg-*5H03	5H_612544142	A/**G**	5H	538,786,402	6.35	0.09	1.07	223
WQ*Rpg-*6H01	6H_576683996	G/**C**	6H	555,502,895	9.08	0.07	2.83	149
WQ*Rpg-*7H01	7H_107066581	C/**A**	7H	102,573,432	8.71	0.11	1.92	1,405
WQ*Rpg-*7H02	7H_448221909	G/**A**	7H	433,462,981	7.48	0.14	0.93	725
WQ*Rpg-*7H03	7H_631891623	**A**/G	7H	604,620,989	6.01	0.05	5.45	133

^a^Bolded alleles correspond to resistance to PNW isolate Lsp21.

^b^Chromosome (Chr) number in which the marker is located.

^c^Physical position of markers according to Morex v3 genome assembly (Mascher et al. 2021).

^d^LOD or log_10_(*P*) of significance associated with resistance.

^e^MAF for each SNP marker.

^f^PVE by individual markers.

The 2 significant MTAs 1H_482689583 and 1H_492949440 (Fig. 3; Table 2) were detected on the long arm of chromosome 1H, with LOD scores of 7.28 and 10.66, respectively. These markers are located at Morex v3 physical positions 449,965,275 and 459,175,825 based on the most recent Morex v3 genome assembly. Linkage decay analysis delimited these loci to 221 and 592 kb regions, designated WQ*Rpg*-1H01 and WQ*Rpg*-1H02, respectively (Table 2).

Chromosome 2H contained the 2 significant MTAs 2H_43141092 and 2H_748987056 (Fig. 3; Table 2), with LOD scores of 7.55 and 6.72, respectively. Marker 2H_43141092 is located on the short arm of chromosome 2H at Morex v3 physical location 37,171,396 bp, and 2H_748987056 is located on the long arm of 2H at physical position 649,620,603 bp based on the Morex v3 genome assembly. The LD decay at MTAs 2H_43141092 and 2H_748987056 delimited the regions to 1,014 and 116 kb, designated the WQ*Rpg*-2H01 and WQ*Rpg*-2H02 loci (Table 2), respectively. However, no high-confidence genes were discovered in WQ*Rpg*-2H02 using the Morex v3 genome assembly. These loci have not been previously reported as stem rust resistance loci.

The MTA 3H_623131530 was the only locus identified on chromosome 3H (Fig. 3; Table 2) and is located at Morex v3 genome position 555,008,110 bp, with a LOD score of 6.07. The delimited region was only 20 kb and designated the WQ*Rpg*-3H01 locus.

Three significant MTAs (5H_435859197, 5H_596832560, and 5H_612544142) were identified on chromosome 5H (Fig. 3; Table 2). The significant MTA 5H_435859197 was located at Morex v3 physical position 388,581,717 bp. This locus was delimited to a large 2 Mbp region and was designated WQ*Rpg*-5H01 (Table 2). The most significant MTA identified in this GWAS analysis, with a LOD score of 11.19, was 5H_596832560, located on the long arm of chromosome 5H at Morex v3 physical position 526,215,093 bp (Fig. 3; Table 2). Furthermore, 36% of the phenotypic variation was explained by this individual marker. This locus was delimited to a 133 kb region designated the WQ*Rpg-*5H02 locus (Table 2). The last significant MTA detected on 5H is 5H_612544142 (Fig. 3; Table 2), located at Morex v3 physical position 538,786,402 bp. This locus was delimited to a 223 kb region designated the WQ*Rpg*-5H03 locus, which contains a high-confidence gene model HORVU.MOREX.r3.5HG0515150.1 using the Morex v3 genome assembly. Interestingly, this gene model encodes an F-box protein (Table 2).

Chromosome 6H contained 1 significant MTA 6H_576683996 (Fig. 3; Table 2). This MTA was located at Morex v3 physical position 555,502,895 bp with a LOD score of 9.06 and was designated the WQ*Rpg*-6H01 locus (Table 2). This locus was delimited to a region of 149 kb in the Morex v3 genome assembly, which contained the high-confidence gene model HORVU.MOREX.r3.6HG0630680.1, a predicted leucine-rich repeat (LRR) protein.

Lastly, chromosome 7H contained the 3 significant MTAs 7H_107066581, 7H_448221909, and 7H_631891623 (Fig. 3; Table 2). The 7H_107066581 MTA is located on the short arm of 7H at Morex v3 physical position 102,573,432 bp, with a LOD score of 8.71. This locus, designated WQ*Rpg*-7H01 (Table 2), was delimited to a large physical region of 1.27 Mb. The second significant marker identified on chromosome 7H was 7H_448221909 (Fig. 3; Table 2), located at Morex v3 physical position 433,462,981 bp with a LOD score of 7.48. This locus, designated WQ*Rpg*-7H02, was delimited to a region of 725 kb. The last and lowest significant marker was 7H_631891623 (Fig. 3; Table 2), with a LOD score of 6.01. Marker 7H_631891623 is at Morex v3 physical position 604,620,989 bp. This locus, designated the WQ*Rpg*-7H03 (Table 2), was delimited to a region of 133 kb.

### Pangenome analysis

No classical disease resistance genes were found in the Morex v3 genome assembly except for WQ*Rpg*-6H01, which included an LRR. However, the lack of candidate genes identified in these regions could be due to the use of the domestic Morex v3 genome assembly as the reference genome as opposed to using the wild pangenome assemblies. Each of these delimiting loci was then compared to the 9 wild genome accessions in the Barley Pangenome v2 (Jayakodi et al. 2024) using nonsignificant flanking markers (Supplementary Tables 5 and 6). For locus WQ*Rpg*-1H01 in the Morex v3 genome assembly, there are only 4 high-confidence genes in a roughly 220 kb region. However, in WBDC-348 and WBDC-349, the region expands to ∼2 Mb physical region containing 27 to 50 high-confidence genes. Furthermore, the WQ*Rpg*-1H01 locus in WBDC-349 is inverted. A leucine repeat protein kinase (HORVU.WBDC349.PROJ.1HG00043210) was discovered in WBDC-349 that was not found in any of the other genome assemblies.

When comparing locus WQ*Rpg*-2H01 to the 9 WBDC assemblies, the number of genes in this region ranged from 11 (WBDC-184 and WBDC-237) to 16 (WBDC-349) (Supplementary Table 6). For locus WQ*Rpg*-2H01, only WBDC-133 contained candidate gene HORVU.WBDC103.PROJ.2HG00071190, which is predicted to encode an LRR receptor-like serine/threonine protein kinase (S/TPK). No other accession contains this gene. The locus identified as WQ*Rpg*-2H02 was delimited to genomic regions based on the Morex v3 genome assembly that did not contain any high-confidence gene models/candidate genes. Interestingly, the accessions WBDC-207 and WBDC-349 were 1.2 and 800 kbp larger than Morex v3. Furthermore, WBDC-207, WBDC-348, and WBDC-349 contained at least 1 high-confidence gene, with WBDC-349 containing 22 high-confidence genes. One of the predicted high-confidence gene models within WBDC-349 was a proline-rich receptor-like protein kinase (HORVU.WBDC349.PROJ.2HG00134960).

Locus WQ*Rpg*-3H01 contained only 1 gene in Morex v3 (Supplementary Table 6). When comparing this locus to the WBDC pangenomes, there was not much difference. Each accession only contained 1 high-confidence gene. All accessions except for WBDC-103 contain an ankyrin repeat-containing protein, which is the same in Morex v3. However, WBDC-103 contained a neurogenic locus notch homolog protein 1 at this location. However, no classical disease resistance genes were found at WQ*Rpg*-3H01.

WQ*Rpg*-5H01 and WQ*Rpg*-5H02 did not contain any disease resistance genes (Supplementary Table 6). However, within the delimited WQ*Rpg*-5H01 locus, there were more predicted genes in the wild accessions compared to Morex. Furthermore, accession WBDC-184 was inverted and contained over 100 additional gene models. The delimited region for locus WQ*Rpg*-5H03 contained an F-box family protein in all accessions except WBDC-103, WBDC-133, and WBDC-207.

For WQ*Rpg*-6H01, 3 accessions WBDC-78, WBDC-103, WBDC-237 had flanking markers translocated to chromosome 3H (Supplementary Table 6). However, within the delimited regions of WBDC-78 and WBDC-103, there was a leucine-rich receptor-like protein kinase (HORVU.WBDC078.PROJ.6HG00398930 and HORVU.WBDC103.PROJ.6HG00395610).

WQ*Rpg*-7H02 Morex v3 only contained 5 genes where the wild accessions expanded from 6 to 18 genes (Supplementary Table 6). In WBDC-78, WBDC-103, WBDC-133, and WBDC-349, there was a S/TPK located within the delimiting region. Lastly, when comparing locus WQ*Rpg*-7H02 to the 9 WBDC assemblies, the number of genes in this region ranged from 5 (Morex v3) to 18 (WBDC-103). Furthermore, there was a S/TPK in WBDC-78, WBDC-103, WBDC-133, and WBDC-348.

## Discussion

Seven of the 12 significant MTAs found in this study (WQ*Rpg*-2H01, WQ*Rpg*-2H02, WQ*Rp*g-3H01, WQ*Rpg*-5H01, WQ*Rpg*-5H03, WQ*Rpg*-7H02, and WQ*Rpg*-7H03) potentially represent novel stem rust resistance loci. Interestingly, 4 MTAs (WQ*Rpg*-1H01, WQ*Rpg*-1H02, WQ*Rpg-*6H01, and WQ*Rpg*-7H03) were delimited to regions with only 1 candidate gene based on the Morex v3 genome assembly. The WQ*Rpg*-1H02 and WQ*Rpg-*6H01 loci were intriguing as they contain high-confidence gene models within the Morex v3 assembly that were predicted to encode a respiratory burst oxidase (RBO)-like protein and an LRR, respectively. These genes have been shown to play direct roles in disease resistance within plants. RBO proteins regulate reactive oxygen species (ROS) production upon pathogen attack, serving as signaling molecules to activate defense genes and programed cell death (Torres et al. 2006; Mittler et al. 2011). The production of ROS can trigger both pathogen-associated molecular pattern triggered immunity (PTI) and effector-triggered immunity (ETI) responses; thus, RBO proteins play an important role in plant defense responses (Zhang and Zhou 2010 ; Kadota et al. 2015). The LRR proteins identified are also notable because they are key recognition modules found across diverse plant immune receptors (Wang et al. 2013; Zhang et al. 2017; Huanhuan et al. 2022; Soltabayeva et al. 2022 ; Wei et al. 2023; Ma et al. 2025). Thus, this GWAS analysis effectively delimited resistance loci to physical regions that contained a single candidate gene, which demonstrates the power of combining robust phenotyping, high-density marker saturation, and modern mapping algorithms. However, due to the expected diversity present between domesticated and wild barley, the delimited physical regions for each locus identified using the Morex v3 reference genome could be expanded or collapsed in the diverse WBDC lines and contain indels and/or translocations containing additional candidate genes. The comparative analysis using wild barley assemblies from the Barley Pangenome v2 determined that WQ*Rpg*-1H01, WQ*Rpg*-2H01, WQ*Rpg*-2H02, WQ*Rpg*-6H01, WQ*Rpg*-7H01, and WQ*Rpg*-7H02 contained differences in genome architecture and additional high-confidence candidate gene models.

The WBDC was previously studied using the diverse *Pgt* races QCCJB, MCCFC, HKHJC, TTKSK, and *Pgs* isolate 92-MN-90 to identify novel sources of stem rust resistance (Sallam et al. 2017; Steffenson et al. 2017; Case et al. 2018). However, none of these races, even the virulent African *Pgt* race TTKSK, have virulence on *Rpg1* and RMRL when stacked together, as shown with several of the PNW *Pgt* isolates (Upadhaya et al. 2022), including the most virulent PNW *Pgt* isolate Lsp21. Lsp21 isolate was chosen for this study to identify novel resistance sources and genetically characterize these loci that are effective against the virulent PNW isolates.

On chromosome 1H, an interval associated with resistance against *Pgt* races MCCFC, TTKSK, and *Pgs* isolate 92-MN-90 was identified between the physical genomic positions 449,965,067 bp (1H_482689791) and 467,954,151 bp (S1H_503256550) using Morex v3 (Sallam et al. 2017; Clare et al. 2024), of which both the WQ*Rpg-*1H01 and WQ*Rpg-*1H02 loci are encompassed within. On chromosome 5H, Sallam et al. (2017) also reported the significant marker S5H_596737839 locus at position 526,348,308 bp (Clare et al. 2024), which is only ∼133 kb away from WQ*Rpg*-5H02 (5H_596832560), the most significant MTA identified in this study. The Sallam_QTL5H-4 locus was associated with resistance against *Pgt* races TTKSK, QCCJB, MCCFC, and *Pgs* isolate 92-MN-90. Two candidate genes were reported for Sallam_QTL5H-4: HORVU5Hr1G094700 and HORVU5Hr1G094710. These genes are predicted to encode disease resistance-responsive dirigent-like proteins, which are the same as the top candidate genes HORVU.MOREX.r3.5HG0510230.1 and HORVU.MOREX.r3.5HG0510240.1 proposed for the WQ*Rpg-*5H02 locus identified in this GWAS. Lastly, Sallam et al. (2017) reported the significant MTA S7H_112969908 on chromosome 7H at position 107,674,001 bp (Clare et al. 2024). This locus is ∼5 Mb from the WQ*Rpg*-7H01 (7H_107066581) locus identified in this study. Based on the LD decay of 1.27 Mb at this region, it is likely that WQ*Rpg*-7H01 is distinct from the 7H region identified by Sallam et al. (2017) and represents a novel resistance locus or gene.

### Candidate genes within identified QTL regions

Comparative analysis of the delimited regions between Morex v3 and wild barley accessions present in the barley pangenome showed a high level of diversity in genome architecture and gene content. Additional candidate genes were identified in wild accessions that are not present in Morex, indicating that wild accessions within the GWAS panel potentially contain additional candidate genes (Supplementary Table 6). Some of the additional candidate genes are predicted to encode typical disease resistance-like proteins, including LRR protein kinase family proteins, suggesting that additional high-priority candidate genes can be mined from the pangenome. From the 8 named stem rust resistance genes in barley, *Rpg1*, *rpg4*, and *Rpg5* have been identified (Brueggeman et al. 2002, Brueggeman et al. 2008; Wang et al. 2013), and all contain STPK domains and fall within known disease resistance-like gene families. *Rpg1* encodes a protein kinase with dual kinase domains, which at the time was a unique plant disease resistance protein structure (Brueggeman et al. 2002; Kleinhof et al. 2009). It was also discovered that *Rpg5* encodes a typical nucleotide-binding site-leucine-rich repeat (NLR) resistance gene yet contains a C-terminal S/TPK-integrated domain. Allele analysis determined that the S/TPK domain is required for pathogen recognition and resistance, and the majority of susceptible alleles contain a protein phosphatase 2C domain in place of the S/TPK. Interestingly, further analysis determined that *Rpg5* is required for *rpg4*-mediated wheat stem rust resistance, yet it is still not fully understood why *Rpg5*-mediated resistance is dominant against rye stem rust yet is recessive against multiple wheat stem rust isolates.

### LD

Based on the LD decay values for each significant SNP, we can delimit the region of interest and identify candidate genes. The average linkage decay calculated in this study was ∼170 bp. LD decay is typically lower in wild barley populations than in domesticated and landrace populations, due to outcrossing events that have occurred over a long evolutionary period, resulting in high levels of recombination (Morrell et al. 2005). Rapid LD decay has been reported in wild barley populations within only a few hundred base pairs (Caldwell et al. 2006; Sallam et al. 2017). Sallam et al. (2017) reported low levels of association between adjacent markers within the 318 accessions of the WBDC. These levels of LD decay are similar to those reported for outbreeding species such as *Zea mays* (Morrell et al. 2005).

### Resistance to Lsp21

This study aimed to identify novel stem rust resistance loci in the WBDC effective against the most virulent *Pgt* isolate ever reported on barley, *Pgt* isolate Lsp21. The PNW *Pgt* population collected in 2019 was shown to be the most virulent *Pgt* population reported worldwide (Upadhaya et al. 2022, 2024). It contains isolates such as Lsp21 that are virulent on barley lines Morex (*Rpg1*+), CIho 7124 (*Rpg2+*), PI282313 (*Rpg3+*), HQ1 (RMRL+), Q21861 (*Rpg1*+ and RMRL+), and Black Hulless (*rpg8+*) (Supplementary Table 2). It was observed that 99% of the 100 isolates collected from the PNW were virulent on Morex, which contains the resistance gene *Rpg1*. Furthermore, 10% of the isolates were virulent on Q21861, which contains both resistance genes *Rpg1* and RMRL. This remarkable virulence is the first documentation of *Pgt* virulence on the *Rpg1/*RMRL gene combination worldwide. The 6 MTAs identified in this GWAS present on chromosomes 5H and 7H did not colocalize with RMRL or *Rpg1*, respectively, and both genes were ineffective against the isolate Lsp21. Thus, we have identified novel resistance genes that do not appear to represent different alleles of RMRL and *Rpg1*. However, 22 lines from the WBDC do contain a function RMRL locus (Sallam et al. 2017; Steffenson et al. 2017).

Twenty-two WBDC accessions showed resistance to the virulent *Pgt* isolate Lsp21. Interestingly, the WBDC accessions containing the newly discovered *R*-gene, *Rpg7*, showed remarkable levels of resistance against *Pgt* isolate Lsp21. *Rpg7* is considered to provide remarkable stem rust resistance in barley because previously identified and characterized barley *R*-genes typically display a lower level of resistance, in line with non-race-specific resistance sources mediating a slow rusting phenotype. *Rpg7* is resistant to *Pgt* races QCCJB, MCCFC, HKHJC, TTKSK, and *Pgs* race 92-MN-90, not to TTKSA, at the adult plant stage (Sallam et al. 2017; Henningsen et al. 2021). Thus, it still displays a broad range of resistance, as demonstrated by many known barley *R-*genes. WBDC-94 and WBDC-238 are the only 2 accessions that contain *Rpg7* within the whole WBDC (Henningsen et al. 2021). Because the frequency of this gene is low in the population, *Rpg7* was not identified in the GWAS as a significant MTA. The inability to pick up MTAs in low frequencies is a common characteristic of a GWAS (Bernardo 2008). Thus, this gene is an excellent candidate for high-resolution biparental mapping and positional cloning based on previous studies showing that the single dominant *Rpg7* confers this remarkable resistance (Henningsen et al. 2021).

### Conclusion

The domestication of wild barley and thousands of years of selection have created a genetic diversity bottleneck in domestic barley. One way to reverse this is the characterization of WBDCs to identify novel genes contributing to traits of interest. Here, we utilized the WBDC and a GWAS approach to identify stem rust resistance loci and candidate genes that are effective against a virulent isolate from the PNW population for which barley has no known resistance. The WBDC GWAS analysis identified 7 novel loci on chromosomes 2H, 3H, 5H, and 7H associated with resistance to *Pgt* isolate Lsp21. However, the previously characterized loci are also of interest as we begin introgressing and stacking these resistances into domesticated elite barley germplasm. Mapping these new sources of stem rust resistance genes in barley is crucial for integrating diverse *R*-genes into elite barley backgrounds to enhance resistance to the virulent PNW *Pgt* population. Lastly, the phenotyping of the WBDC identified the remarkable resistance presumably conferred by the *Rpg7* gene. Thus, the next step is the positional cloning and identification of *Rpg7* via high-resolution biparental mapping, which is currently underway.

## Supplementary Material

jkaf300_Supplementary_Data

## Data Availability

The authors affirm that all data necessary to confirm the article's conclusions are in the manuscript and supplementary files. Furthermore, the complete genotype data for all WBDC accessions can be found on T3/Barley website at https://barley.triticeaetoolbox.org/breeders/trial/1653?format=. Supplemental material available at G3 online.

## References

[jkaf300-B1] Barrett JC, Fry B, Maller J, Daly MJ. 2005. Haploview: analysis and visualization of LD and haplotype maps. Bioinformatics. 21:263–265. 10.1093/bioinformatics/bth457. 15297300

[jkaf300-B2] Beier S et al 2017. Construction of a map-based reference genome sequence for barley, Hordeum vulgare L. Sci Data. 4:170044. 10.1038/sdata.2017.44. 28448065 PMC5407242

[jkaf300-B3] Bernardo R . 2008. Molecular markers and selection for complex traits in plants: learning from the last 20 years. Crop Sci. 48:1649–1664. 10.2135/cropsci2008.03.0131.

[jkaf300-B4] Blum M et al 2021. The InterPro protein families and domains database: 20 years on. Nucleic Acids Res. 49:D344–D354. 10.1093/nar/gkaa977. 33156333 PMC7778928

[jkaf300-B5] Bradbury PJ et al 2007. TASSEL: software for association mapping of complex traits in diverse samples. Bioinformatics. 23:2633–2635. 10.1093/bioinformatics/btm308. 17586829

[jkaf300-B6] Brooke M, Upadhaya A, Clare S, Brueggeman R. 2025. Quantitative trait loci analysis of a novel source of barley seedling resistance effective against the virulent North American stem rust pathogen. Phytopathology. 115:724–732. 10.1094/PHYTO-07-24-0231-R. 39961036

[jkaf300-B7] Brueggeman R, et al 2008. The stem rust resistance gene *Rpg5* encodes a protein with nucleotide-binding-site, leucine-rich, and protein kinase domains. Proc Natl Acad Sci. 105:14970–14975. 10.1073/pnas.0807270105. 18812501 PMC2567477

[jkaf300-B8] Brueggeman R, Rostoks N, Kleinhofs A. 2002. The barley stem rust-resistance gene *Rpg1* is a novel disease-resistance gene with homology to receptor kinases. Proc Natl Acad Sci. 99:9328–9333. 10.1073/pnas.142284999. 12077318 PMC123140

[jkaf300-B9] Caldwell KS, Russell J, Langridge P, Powell W. 2006. Extreme population-dependent linkage disequilibrium detected in an inbreeding plant species, Hordeum vulgare. Genetics. 172:557–567. 10.1534/genetics.104.038489. 16219791 PMC1456183

[jkaf300-B10] Case AJ et al 2018. Mapping adult plant stem rust resistance in barley accessions Hietpas-5 and GAW-79. Theor Appl Genet. 131:2245–2266. 10.1007/s00122-018-3149-8. 30109391

[jkaf300-B11] Clare SJ et al 2023. Wild barley (*Hordeum spontaneum*) and landraces (*Hordeum vulgare*) from Turkey contain an abundance of novel Rhynchosporium commune resistance loci. Theor Appl Genet. 136:15. 10.1007/s00122-023-04245-w. 36662256

[jkaf300-B12] Clare SJ, Novakazi F, Hayes PM, Moscou MJ, Brueggeman RS. 2024. Colocalization of genetic regions that confer resistance/susceptibility against Puccinia species and association with Pyrenophora teres loci within the barley genome. Front Agron. 6:1451281. 10.3389/fagro.2024.1451281.

[jkaf300-B13] Dyck PL, Kerber ER. 1985. Resistance of the race-specific type. In: Roelfs Alan P, Bushnell William R, editors. The cereal rusts: diseases, distribution, epidemiology, and control. vol. II. Academic Press. p. 469–500. 10.1016/C2013-0-10449-7.

[jkaf300-B14] Ellis RP et al 2000. Wild barley: a source of genes for crop improvement in the 21st century? J Exp Bot. 51:9–17. 10.1093/jexbot/51.342.9. 10938791

[jkaf300-B15] Fetch TG, Steffenson BJ, Nevo E. 2003. Diversity and sources of multiple disease resistance in *Hordeum spontaneum*. Plant Dis. 87:1439–1448. 10.1094/PDIS.2003.87.12.1439. 30812385

[jkaf300-B16] Henningsen E et al 2021. *Rpg7* : a new gene for stem rust resistance from *Hordeum vulgare* ssp. *spontaneum*. Phytopathology. 111:548–558. 10.1094/PHYTO-08-20-0325-R. 32880513

[jkaf300-B17] Hernandez J et al 2019. Introgression of *rpg4*/*Rpg5* into barley germplasm provides insights into the genetics of resistance to *Puccinia graminis* f. sp. *tritici* race TTKSK and resources for developing resistant cultivars. Phytopathology. 109:1018–1028. 10.1094/PHYTO-09-18-0350-R. 30714882

[jkaf300-B18] Hill SR . 2003. Conservation assessment for American barberry (Berberis canadensis Mill.). Ill Nat Hist Surv Tech Rep. [published online ahead of print] [accessed 2024 May 22]. https://www.ideals.illinois.edu/items/10400/bitstreams/38020/data.pdf.

[jkaf300-B19] Huang M, Liu X, Zhou Y, Summers RM, Zhang Z. 2019. BLINK: a package for the next level of genome-wide association studies with both individuals and markers in the millions. GigaScience. 8:giy154. 10.1093/gigascience/giy154. 30535326 PMC6365300

[jkaf300-B20] Huanhuan Y et al 2022. The Sm gene conferring resistance to gray leaf spot disease encodes an NBS-LRR (nucleotide-binding site-leucine-rich repeat) plant resistance protein in tomato. Theor Appl Genet. 135:1467–1476. 10.1007/s00122-022-04047-6. 35165745

[jkaf300-B21] Jayakodi M et al 2024. Structural variation in the pangenome of wild and domesticated barley. Nature. 636:654–662. 10.1038/s41586-024-08187-1. 39537924 PMC11655362

[jkaf300-B22] Jin Y, Rouse M, Groth J. 2014. Population diversity of *Puccinia graminis* is sustained through sexual cycle on alternate hosts. J Integr Agric. 13:262–264. 10.1016/S2095-3119(13)60647-4.

[jkaf300-B23] Kadota Y, Shirasu K, Zipfel C. 2015. Regulation of the NADPH oxidase RBOHD during plant immunity. Plant Cell Physiol. 56:1472–1480. 10.1093/pcp/pcv063. 25941234

[jkaf300-B24] Kleinhofs A, Brueggenman R, Nirmala J, Zhang L, Mirlohi A, Druka A, Rostoks N, Steffenson B. 2009. Barley stem rust resistance genes: structure and function. Plant Genome. 2. 10.3835/plantgenome2009.02.0011.

[jkaf300-B25] Lipka AE et al 2012. GAPIT: genome association and prediction integrated tool. Bioinformatics. 28:2397–2399. 10.1093/bioinformatics/bts444. 22796960

[jkaf300-B26] Liu M et al 2020. The draft genome of a wild barley genotype reveals its enrichment in genes related to biotic and abiotic stresses compared to cultivated barley. Plant Biotechnol J. 18:443–456. 10.1111/pbi.13210. 31314154 PMC6953193

[jkaf300-B27] Ma S et al 2025. OsBRW1, a novel blast-resistant gene, coded a NBS-LRR protein to interact with OsSRFP1 to balance rice growth and resistance. Plant Biotechnol J. 23:250–267. 10.1111/pbi.14494. 39492591 PMC11672734

[jkaf300-B28] Maloy OC . 1993. Plant disease control: principles and practice. John Wiley & Sons. p. 346.

[jkaf300-B29] Mascher M et al 2021. Long-read sequence assembly: a technical evaluation in barley. Plant Cell. 33:1888–1906. 10.1093/plcell/koab077. 33710295 PMC8290290

[jkaf300-B30] Matny O et al 2024. Molecular mapping of the stem rust resistance gene *rpg8* in barley. Phytopathology. 114:109–109. 10.13140/RG.2.2.30912.75522.

[jkaf300-B31] McKay R . 1957. Cereal diseases in Ireland. Arthur Ginness, Son.

[jkaf300-B32] Miller JD, Lambert JW. 1955. Variability and inheritance of reaction of barley to race 15B of stem rust. Agron J. 47:373–377.

[jkaf300-B33] Mittler R et al 2011. ROS signaling: the new wave? Trends Plant Sci. 16:300–309. 10.1016/j.tplants.2011.03.007. 21482172

[jkaf300-B34] Morrell PL, Toleno DM, Lundy KE, Clegg MT. 2005. Low levels of linkage disequilibrium in wild barley (*Hordeum vulgare* ssp. *spontaneum*) despite high rates of self-fertilization. Proc Natl Acad Sci U S A. 102:2442–2447. 10.1073/pnas.0409804102. 15699350 PMC549024

[jkaf300-B35] Pook T et al 2020. Improving imputation quality in BEAGLE for crop and livestock data. G3 (Bethesda). 10:177–188. 10.1534/g3.119.400798. 31676508 PMC6945036

[jkaf300-B36] Roelfs AP . 1978. Estimated losses caused by rust in small grain cereals in the United States, 1918–76. Department of Agriculture, Agricultural Research Service.

[jkaf300-B37] Roelfs AP . 1982. Effects of barberry eradication. Plant Dis. 66:177. 10.1094/PD-66-177. 30786626

[jkaf300-B38] Roelfs AP . 1985. Wheat and rye stem rust. In: Roelfs Alan P, Bushnell William R, editors. The Cereal Rusts: Diseases, distribution, epidemiology, and control. vol. II. Academic Press. p. 3–37. 10.1016/C2013-0-10449-7.

[jkaf300-B39] Sallam AH et al 2017. Genome-wide association mapping of stem rust resistance in *Hordeum vulgare* subsp. *spontaneum*. G3 (Bethesda). 7:3491–3507. 10.1534/g3.117.300222. 28855281 PMC5633397

[jkaf300-B40] Soltabayeva A et al 2022. Receptor-like kinases (LRR-RLKs) in response of plants to biotic and abiotic stresses. Plants. 11:2660. 10.3390/plants11192660. 36235526 PMC9572924

[jkaf300-B41] Stakman EC, Fletcher DG. 1930. The common barberry and black stem rust. U.S. Department of Agriculture.

[jkaf300-B42] Stakman EC, Stewart DM, Loegering WQ. 1962. Identification of physiologic races of Puccinia graminis var. tritici. Identif Physiol Races Puccinia Graminis Var Tritici. [published online ahead of print].

[jkaf300-B43] Steffenson BJ et al 2007. A walk on the wild side: mining wild wheat and barley collections for rust resistance genes. Aust J Agric Res. 58:532–544. 10.1071/AR07123.

[jkaf300-B44] Steffenson BJ et al 2017. Vulnerability of barley to African pathotypes of *Puccinia graminis* f. sp. *tritici* and sources of resistance. Phytopathology. 107:950–962. 10.1094/PHYTO-11-16-0400-R. 28398875

[jkaf300-B45] Torres MA, Jones JDG, Dangl JL. 2006. Reactive oxygen species signaling in response to pathogens. Plant Physiol. 141:373–378. 10.1104/pp.106.079467. 16760490 PMC1475467

[jkaf300-B46] Upadhaya A . 2023. Genetic characterization of virulence in a Pacific Northwest stem rust population and mapping of new sources of resistance in barley. Washington State University.

[jkaf300-B47] Upadhaya A, Upadhaya SG, Brueggeman R. 2022. The wheat stem rust (*Puccinia graminis* f. sp. *tritici*) population from Washington contains the most virulent isolates reported on barley. Plant Dis. 106:223–230. 10.1094/PDIS-06-21-1195-RE. 34546770

[jkaf300-B48] Upadhaya A, Upadhaya SG, Brueggeman R. 2024. Identification of candidate avirulence and virulence genes corresponding to stem rust (*Puccinia graminis* f. sp. *tritici*) resistance genes in wheat. Mol Plant-Microbe Interact. 37:635–649. 10.1094/MPMI-05-24-0056-R. 38780476

[jkaf300-B49] von Bothmer R et al 2003. The domestication of cultivated barley. Elsevier. [accessed 2024 May 21].

[jkaf300-B50] Wambugu PW, Ndjiondjop M-N, Henry RJ. 2018. Role of genomics in promoting the utilization of plant genetic resources in genebanks. Brief Funct Genomics. 17:198–206. 10.1093/bfgp/ely014. 29688255 PMC5967547

[jkaf300-B51] Wang X et al 2013. The *rpg4* -mediated resistance to wheat stem rust (*Puccinia graminis*) in barley (*Hordeum vulgare*) requires *Rpg5*, a second NBS-LRR gene, and an actin depolymerization factor. Mol Plant-Microbe Interact. 26:407–418. 10.1094/MPMI-06-12-0146-R. 23216085

[jkaf300-B52] Wang J, Zhang Z. 2021. GAPIT version 3: boosting power and accuracy for genomic association and prediction. Genomics Proteomics Bioinformatics. 19:629–640. 10.1016/j.gpb.2021.08.005. 34492338 PMC9121400

[jkaf300-B53] Wei W et al 2023. An NBS-LRR protein in the Rpp1 locus negates the dominance of Rpp1-mediated resistance against Phakopsora pachyrhizi in soybean. Plant J. 113:915–933. 10.1111/tpj.16038. 36424366

[jkaf300-B54] Yao E et al 2022. GrainGenes: a data-rich repository for small grains genetics and genomics. Database. 2022:baac034. 10.1093/database/baac034. 35616118 PMC9216595

[jkaf300-B55] Yin L et al 2021. rMVP: a memory-efficient, visualization-enhanced, and parallel-accelerated tool for genome-wide association study. Genomics Proteomics Bioinformatics. 19:619–628. 10.1016/j.gpb.2020.10.007. 33662620 PMC9040015

[jkaf300-B56] Zhang C et al 2017. Overexpression of a novel peanut NBS-LRR gene AhRRS5 enhances disease resistance to Ralstonia solanacearum in tobacco. Plant Biotechnol J. 15:39–55. 10.1111/pbi.12589. 27311738 PMC5253469

[jkaf300-B57] Zhang J, Zhou J-M. 2010. Plant immunity triggered by microbial molecular signatures. Mol Plant. 3:783–793. 10.1093/mp/ssq035. 20713980

[jkaf300-B58] Zhou H et al 2014. Association mapping of stem rust race TTKSK resistance in US barley breeding germplasm. Theor Appl Genet. 127:1293–1304. 10.1007/s00122-014-2297-8. 24710821 PMC4035542

