# Supplementary material for: Identification and association mapping of sources of stem rust resistance in the wild barley diversity panel effective against virulent isolates from the Pacific Northwest
Source: G3 (Bethesda). 2025 Dec 20;16(3):jkaf300. doi: 10.1093/g3journal/jkaf300 (PMC12958803; doi:10.1093/g3journal/jkaf300)
Supplement: jkaf300_Supplementary_Data [file jkaf300_supplementary_data.zip › Supplementary_Material_Legends_G3-2025-405973.docx]

Supplementary Table S1: The list of 318 Wild Barley Diversity Collection (WBDC), *Hordeum vulgare* subsp. *spontaneum* accessions, obtained from the USDA National Small Grains Collection, Aberdeen, ID

Supplementary Table S2: Five stem rust isolates from the inland northwest and barley and wheat's resistance and susceptibility reactions (Data derived from Upadhaya et al., 2022).

Supplementary Table S3: Coefficient of infection scores for 277 WBDC and checks against the virulent PNW *Pgt* isolate Lsp21

Supplementary Table S4: GWAS GAPIT results using BLINK model

Supplementary Table S5: Twelve significant QTL using the BLINK model with the non-significant flanking markers outside the calculated LD blocks to delimit the regions of each MTA

Supplementary Table S6: Summary of the comparative analysis of the delimited locus using the wild barley accession within the Pangenome v2

Supplementary Figure S1: Genome-wide GBS marker density across the seven barley chromosomes.

Supplementary Figure S2: QQ plot comparing the observed versus expected −log₁₀(*p*) values for the CI trait.
