# Supplementary figures and images for: Identification and association mapping of sources of stem rust resistance in the wild barley diversity panel effective against virulent isolates from the Pacific Northwest
Source: G3 (Bethesda). 2025 Dec 20;16(3):jkaf300. doi: 10.1093/g3journal/jkaf300 (PMC12958803; doi:10.1093/g3journal/jkaf300)

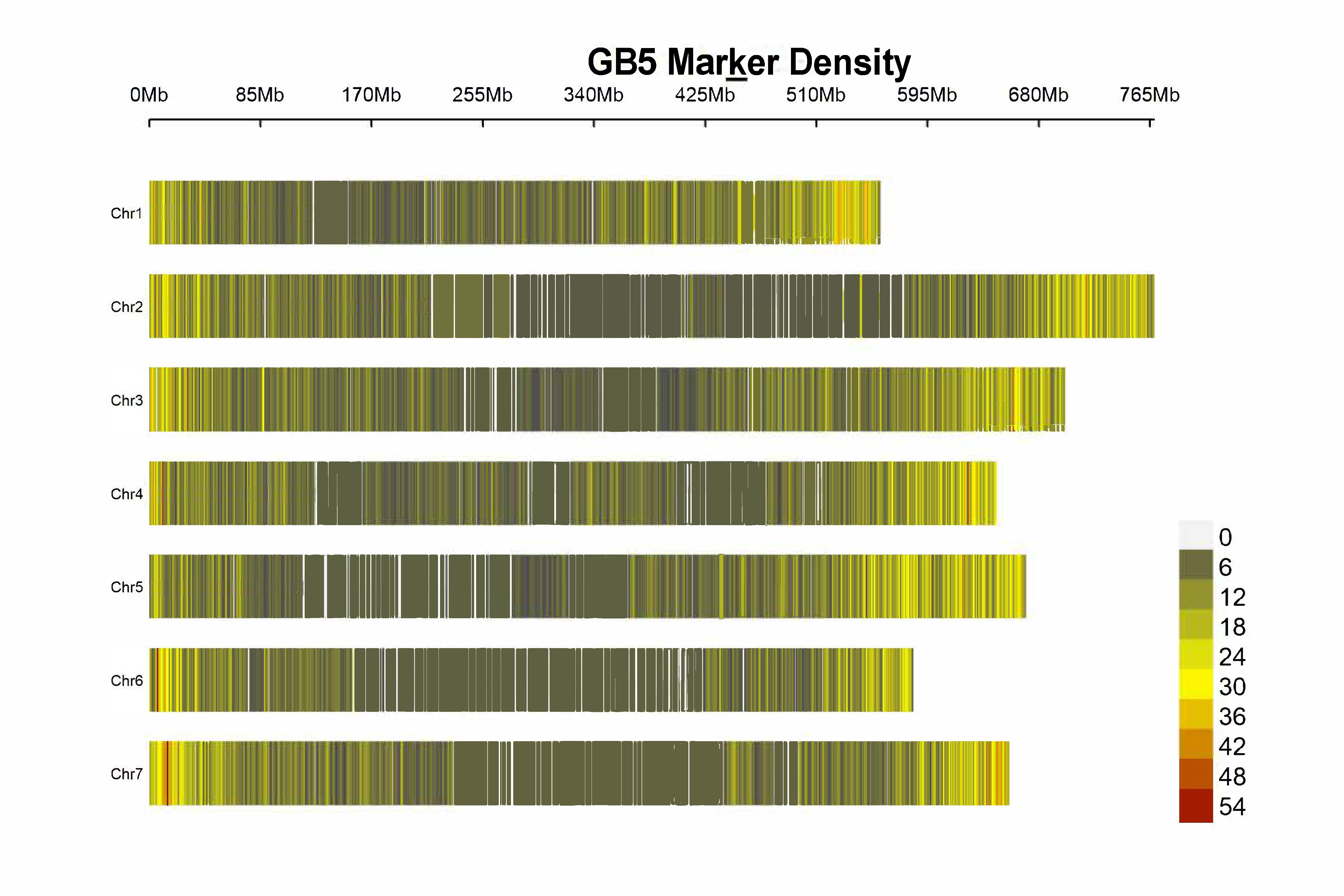

Supplement: jkaf300_Supplementary_Data [file jkaf300_supplementary_data.zip › Supplementary_Figure_S1_G3-2025-405973.png]

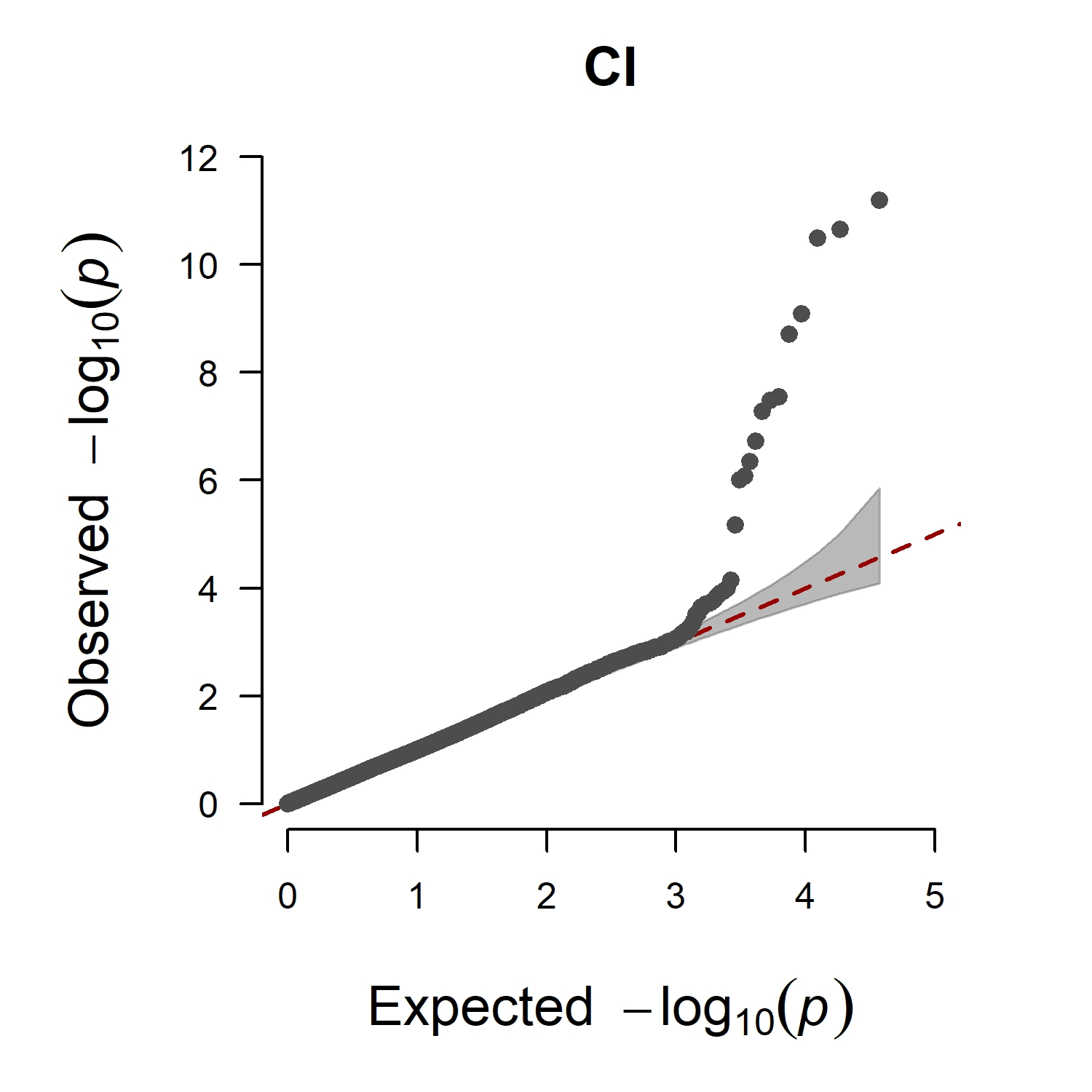

Supplement: jkaf300_Supplementary_Data [file jkaf300_supplementary_data.zip › Supplementary_Figure_S2_G3-2025-405973.png]
